# Supplementary material for: The Role of Imaging in Cervical Cancer Staging: ESGO/ESTRO/ESP Guidelines (Update 2023)
Source: Cancers (Basel). 2024 Feb 14;16(4):775. doi: 10.3390/cancers16040775 (PMC10886638; doi:10.3390/cancers16040775)
Supplement: Supplementary file 1 [file cancers-16-00775-s001.zip › Supplementary Table S1..pdf]

**FIGO 2009**	**FIGO 2018**
Staging was based primarily on clinical findings; in addition, plain radiographs, including intravenous pyelography, can be used for staging.	All imaging modalities such as ultrasound, CT, MRI, or PET-CT and pathologic findings can be used to complement clinical evaluation in assessing both tumour size and extent. The method used to assign the stage should be recorded. Pathologic findings take precedence over clinical assessment and imaging findings in assigning the FIGO stage.
**Stage IA**	**Stage IA**
The classification of IA stage depended on both the extent of horizontal spread and the depth of disease invasion.	The horizontal dimension of the lesion is no longer considered in defining the upper boundary of IA stage.
IA Carcinoma with maximum depth of invasion ≤5 mm with a horizontal spread 7.0 mm of less.	IA Carcinoma with maximum depth of invasion ≤5 mm.
**Stage IB**	**Stage IB**
	The creation of new subcategory (IB3) enables more consistent contribution of tumour size to prognosis. In addition, tumour size cut-off value of 2 cm enables to evaluate potential candidates for fertility-sparing treatment.
IB1 Clinically visible lesion ≤ 4.0 cm in greatest dimension.	IB1 Invasive carcinoma with >5 mm depth of stromal invasion and ≤2 cm in greatest dimension.
IB2 Clinically visible lesion > 4.0 cm in greatest dimension.	IB2 Invasive carcinoma >2 cm and ≤4cm in greatest dimension.
	IB3 Invasive carcinoma >4 cm in greatest dimension
**Stage III**	**Stage III**
The tumour extends to the pelvic wall and/or involves lower third of the vagina and/or causes hydronephrosis or non-functioning kidney. The lymph node status is not incorporated in stage III.	The carcinoma involves the lower third of the vagina and/or extends to the pelvic wall and/or causes hydronephrosis or non-functioning kidney and/or involves pelvic and/or paraaortic lymph nodes.
IIIA Tumour involves the lower third of the vagina, with no extension to the pelvic wall.	IIIA The carcinoma involves the lower third of the vagina, with no extension to the pelvic wall.
IIIB Extension to the pelvic wall and/or hydronephrosis or non-functioning kidney.	IIIB Extension to the pelvic wall and/or hydronephrosis or non-functioning kidney (unless known to be due to another cause).
	IIIC Involvement of pelvic and/or paraaortic lymph nodes (including micrometastasis), irrespective of tumour size and extent
	IIIC1 Pelvic lymph node metastasis only.
	IIIC2 Paraaortic lymph node metastasis.

Supplementary Table S1 Key updates to the 2018 FIGO classification compared to the prior version[9,26].

CT, computerised tomography; MRI, magnetic resonance imaging; PET-CT, positron emission tomography in combination with CT.
