# Supplementary material for: The Role of Imaging in Cervical Cancer Staging: ESGO/ESTRO/ESP Guidelines (Update 2023)
Source: Cancers (Basel). 2024 Feb 14;16(4):775. doi: 10.3390/cancers16040775 (PMC10886638; doi:10.3390/cancers16040775)
Supplement: Supplementary file 1 [file cancers-16-00775-s001.zip › Supplementary Table S2..pdf]

Supplementary Table S2 Ultrasound checklist on cervical cancer

Ultrasound parameter	Description of cervical cancer
**Position of uterus**	▪ *Anteverted / retroverted / upright position*
**Size of the uterus (CC/AP/LL,milimeters)**	▪ ____/____/____
**Size of the uterine cervix (CC/AP/LL,milimeters)**	▪ ____/____/____
**Tumor identification**	▪ *Yes/No.*<br>*If yes,*<br>*assess tumor echogenicity (uniform [hypo- / hyper- / isoechogenic] / non-uniform)*<br>*and perfusion using Color Score*[§]*:* 1 / 2 / 3 / 4
**Tumor site of origin**	▪ *Endocervical*<br>▪ *Ectocervical*<br>▪ *Combined*
**Localisation of tumor within cervix**	▪ *Ventral / dorsal part of cervix*<br>▪ *Lateral right / left*<br>▪ *Global*
**Tumor size (CC/AP/LL, millimetres)**	▪ ____/____/____
**Depth of stromal invasion**	*Proportion of infiltrated stroma to the whole cervix:*<br>▪ *< 50%*<br>▪ *50-75%*<br>▪ *>75%*<br>▪ *The whole cervix*
**Lateral tumor-free distance (milimeters)**[#]	*Minimum diameter between the tumour and pericervical fascia measured between the tumor and the points where ventral, lateral and dorsal parametria attach to the cervix*[#]*:*<br><br>▪ *Ventral right* _____*/ left* ______<br>▪ *Lateral right*_____ */ left* ______<br>▪ *Dorsal right* _____*/ left* ______<br>▪ *Non-applicable (if parametrial invasion in LACC)*
**Cranial tumor-free margin (milimeters)**	▪ *Minimum distance from the upper margin of the tumor to the internal os*:* ______<br>▪ *Non-applicable (if infiltration of isthmus)*
**Infiltration of the uterine isthmus**	▪ *Yes / No.*
**Infiltration of vagina**	▪ *Yes / No. If yes, upper two thirds / lower third.*
**Parametrial involvement**	▪ *Yes /No.*<br>*If yes, site (ventral, lateral, dorsal) and laterality of involved parametrium, including depth and width of infiltration.*<br>*Ventral right grade*____*, width* ____*/depth* ____ *(mm)*<br>*Ventral left grade* ____*, width* ____*/ depth* ____ *(mm)*<br>*Lateral right grade* ____*, width* ____*/depth* ____ *(mm)*<br>*Lateral left grade* ____*, width* ____*/ depth* ____ *(mm)*<br>*Dorsal right grade* ____*, width* ____*/depth* ____ *(mm)*<br>*Dorsal left grade* ____*, width* ____*/ depth* ____ *(mm)*<br><br>*Parametrial invasion grading [45]:*<br>▪ *Grade 0: Intact pericervical fascia*<br>▪ *Grade 1: Disrupted pericervical fascia, no involvement of parametrium*<br>▪ *Grade 2: Incipient infiltration of parametria usually in depth ≤5mm*<br>▪ *Grade 3: Nodular infiltration of parametrium*<br>▪ *Grade 4: Discontinuous parametrial involvement (metastatic paracervical visceral LNs)*<br><br>*If the pelvic side wall is affected, document the involved structures and laterality.***
**Bladder and rectal invasion**	*Sliding sign positive / negative*<br>▪ *Positive sliding sign (the tumor slides over the bladder or the rectum)*

	▪ *Negative sliding sign (tumor is fixed against bladder and/or rectum)*<br><br>*Any bladder and/or rectal involvement present/absent. If yes, grading[45]:*<br>▪ *Grade 0:*<br>*Intact echogenic layer of fibrous and fat tissue between the bladder and/or the rectum and vagina and/or the cervix.*<br>▪ *Grade 1: Disruption of the echogenic outer layer of the bladder and/or rectum but no other signs of invasion.*<br>▪ *Grade 2: Disruption of the hyperechogenic muscle layer but no abnormalities of the inner wall architecture.*<br>▪ *Grade 3: Disruption of all layers with intraluminal tumor spread.*<br><br>*If bladder trigone involvement check:*<br>▪ *Ureteric infiltration right: present / absent*<br>▪ *Ureteric infiltration left: present / absent*
**Hydronephrosis**	▪ *Yes, No.*<br>*If yes, the grade of renal dilation[45]:*<br>▪ *Grade 1: dilation of renal sinus*<br>▪ *Grade 2: dilation of renal sinus and calyces*<br>▪ *Grade 3: sacciform dilation of renal sinus and calyces accompained by renal parenchyma atrophy*
**Regional (pelvic and lumbar (paraaortic) lymph nodes**	*Description of site (external, internal, common iliac; infra-/supramesenteric paraaortic), laterality, number, LN grading (see below):*<br>*Assessment by standardized* ***VITA terms*** *using the classification LN1 –LN5[77]:*<br>▪ *LN1: Normal finding*<br>▪ *LN2: Benign finding*<br>▪ *LN3: Indeterminate, probably benign finding*<br>▪ *LN4: Probably malignant finding*<br>▪ *LN5: Malignant finding*
**Distant spread**	▪ *Distant lymph nodes (site, number, laterality if appropriate, lymph node status LN1-LN5, see above)*<br>▪ *Other distant spread (peritoneal spread, visceral organ metastases and others)*
**Other findings**	▪ *Related / unrelated gynecological / non-gynecological pathologies (i.e., deep venous thrombosis)*
**Staging system**	▪ *TNM and FIGO staging system [15,11]*<br>▪ *Comments and recommendations to additional diagnostic tests to the referring specialist and to multidisciplinary team meeting*

§Color score following IOTA (International Ovarian Tumor Analysis) terms and definitions (Color Score 1, no perfusion; Color Score 2, minimal perfusion; Color Score 3, moderate flow; Color Score 4, highly vascularized .
**For consideration of fertility sparing treatment the minimum cranial tumor-free distance ought to be ≥10 mm.*
# The parameter is evaluated only if absent parametrial invasion and measured in points where ventral, lateral and dorsal parametria attach to the cervix [22].
*** Pelvic side wall is defined as the parietal muscles of the lesser pelvis (Internal obturator, coccygeus, and piriformis muscle), fascia, neurovascular structures, or skeletal portions of the bony pelvis.*
CC/AP/LL, maximum cranio-caudal, antero-posterior, latero-lateral diameter.

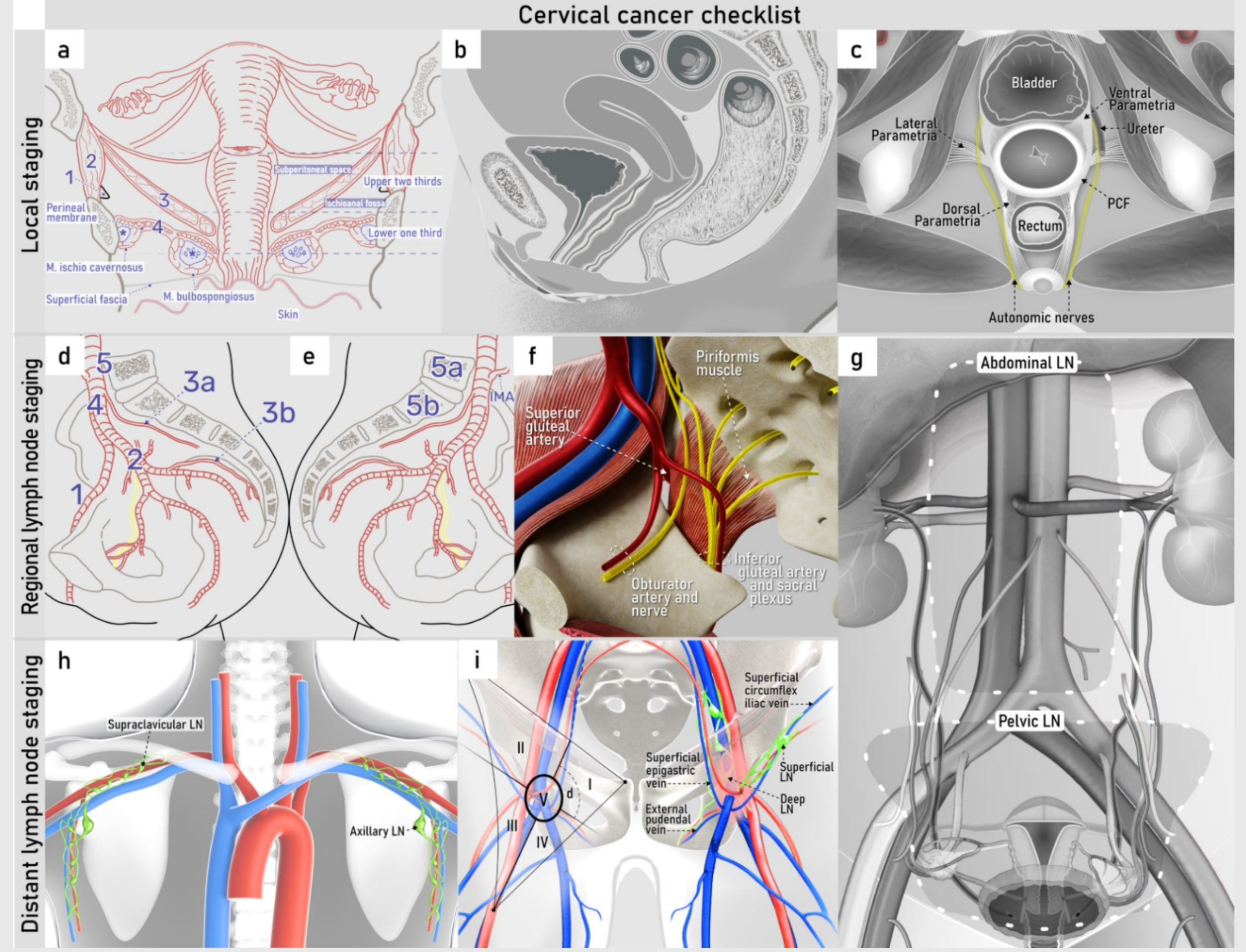

Figure S1 Schematic documentation of cervical cancer staging by ultrasound. *Ultrasound documents the location and extension of primary tumor (local staging, a-c), and any suspicious lymph nodes (size of lymph node and intranodal metastasis, the number of lymph nodes involved, the presence or absence of extracapsular spread and others) (d-g). For local staging, schematics showing the coronal (a), 1 - pudendal (Alcocks canal), 2- obturator internal muscle, 3- levator ani muscle, 4 – deep transverse perineal muscle (transversus perinei profundus); sagittal (b); and axial (c) views of pelvic anatomy. The regional pelvic lymph nodes can be plotted on a diagram of the right (d) and left (e) iliac vessels with the corresponding anatomical diagram (f). In scheme d, 1-external iliac artery, 2- internal iliac artery, 3a-median sacral artery, 3b-lateral sacral artery (to demonstrate drainage for lateral sacral and presacral lymph nodes), 4- common iliac artery and 5- aorta. In scheme e, IMA -inferior mesenteric artery, 5a- supramesenteric region; 5b-inframesenteric region. The regional pelvic and abdominal lymph nodes are delineated by the dashed line in scheme (g). Distant lymph nodes (supraclavicular (scalene) and inguinofemoral lymph nodes) are demonstrated in diagrams (h, i).*
