# Supplementary material for: The Role of Imaging in Cervical Cancer Staging: ESGO/ESTRO/ESP Guidelines (Update 2023)
Source: Cancers (Basel). 2024 Feb 14;16(4):775. doi: 10.3390/cancers16040775 (PMC10886638; doi:10.3390/cancers16040775)
Supplement: Supplementary file 1 [file cancers-16-00775-s001.zip › Supplementary Table S3.pdf]

Study and publication year	Local staging (Yes/No)	LN staging (Yes/No)	Imaging modality	*Tot n° studies*	Outcomes (pooled sensitivity [specificity]%)	Weakness of the study
Choi et al., 2010[84]	No	Yes	Conventional MRI, CT, PET/PET-CT	41	Assessing lymph node metastasis (patient-based):<br>PET/PET-CT 82 [81]<br>MRI 56 [71]<br>CT 50 [92]<br><br>Assessing lymph node metastasis (region/node-based):<br>PET/PET-CT 54 [97]<br>CT 52 [92]<br>MRI 38 [97]	Articles selected from 1981<br><br>Does not categorize early vs LACC<br><br>Nodal regions subgroup analysis not performed
Kang et al.,2010[86]	No	Yes	PET/CT	10	Assessing lymph node metastasis:<br>34 [97]	Does not categorize early vs LACC<br><br>Large heterogeneity of studies<br><br>Paraaortic LN only
Shen et al., 2015[78]	No	Yes	DWI	15	Assessing lymph node metastasis:<br>86 [84]	Most of study designs not documented<br><br>Reference standard not always met (histopathology)<br><br>Pelvic LN only
Liu et al., 2017[92]	No	Yes	Conventional MRI, DWI, CT, PET/PET-CT	67	Assessing lymph node metastasis:<br>DWI 87 [83]<br>PET/PET-CT 66 [97]<br>CT 57 [91]<br>MRI 54 [93]<br><br>Paraaortic LN:<br>PET/PET-CT 81[98]<br>CT 68[90]<br>MRI 54[94]<br>DWI NA<br><br>Pelvic LN:<br>PET/PET-CT 55[97]<br>CT 48[91]<br>MRI 62[93]<br>DWI NA	Study selection from 1981<br><br>Technological features insufficiently detailed
Gong et al.2017[96]	No	Yes	Pelvic conventional MRI, DWI, CT, PET, PET-CT, US	80	Assessing lymph node metastasis:<br>DWI 84 [95]<br>US 71 [99]<br>PET-CT 68 [97]<br>PET 56 [97]<br>MRI 50 [95]<br>CT 47 [93]	Article selected from 1981<br><br>Different gynaecological cancers considered, not cervical exclusive<br><br>Majority of studies being retrospective

						Majority of studies focusing only on pelvic LN<br><br>Nodal regions subgroup analysis not performed<br><br>Reference standard not always met (histopathology)
Woo et al.,2018[95]	Yes	No	Pelvic conventional MRI, DWI	14	Assessing parametrial involvement:<br>DWI 82 [94]<br>MRI 72[91]	Does not categorize early vs LACC<br><br>Different MRI technology (1T;1,5T; 3T)<br><br>Majority of studies being retrospective
Luo et al.,2018[108]	No	Yes	Pelvic conventional MRI, DWI, CT, PET	16	Assessing lymph node metastasis<br>MRI 54[92]<br>PET 50 [96]<br>CT 44 [93]<br>DWI NA*	Does not categorize early vs LACC<br><br>Nodal regions subgroup analysis not performed<br><br>Technological features insufficiently detailed<br><br>*DWI results expressed only in LHR
Ruan et al., 2018[93]	No	Yes	PET/CT	27	Assessing lymph node metastasis:<br>72 [96]<br><br>Paraaortic LN:<br>76 [96]<br><br>Pelvic LN:<br>85 [76]	Inconsistencies in criteria of interpretation of PET/CT<br><br>Large heterogeneity of studies<br><br>Majority of studies being retrospective
Yu et al., 2019[97]	No	Yes	PET/CT	14	Assessing lymph node metastasis:<br>71 [97]	Does not categorize early vs LACC<br><br>Majority of studies being retrospective<br><br>Paraaortic LN only<br><br>Technological features insufficiently detailed

Woo et al.,2020[66]	Yes	Yes	Pelvic conventional MRI, CT, PET, US	115	Assessing local disease extent:<br>PET 73 [91]<br>MRI 71 [91]<br>US 67 [94]<br>CT 43 [71]<br><br>Assessing lymph node metastasis:<br>PET 57 [95]<br>MRI 57 [93]<br>CT 51 [87]<br>US 43 [96]<br><br>Paraaortic LN:<br>PET 59 [96]<br>MRI 40 [91]<br>CT 29 [91]<br>US NA<br><br>Pelvic LN:<br>PET 60 [93]<br>MRI 61 [88]<br>CT NA<br>US NA	Does not categorize early vs LACC<br><br>Majority of studies being retrospective<br><br>Technological features insufficiently detailed
Alcázar et al., 2020[73]	Yes	No	Pelvic conventional MRI, US	9	Assessing parametrial involvement:<br>US 78 [96]<br>MRI 68 [91]	Articles selected from 1990<br><br>Different MRI technologies (0,5 T; 1,5T; 3T; NA)<br><br>Large heterogeneity of studies
Xiao et al., 2020 [65]	Yes	Yes	Conventional MRI	39	Assessing the internal os involvement:<br>86 [97]<br><br>Assessing the stromal invasion:<br>87 [91]<br><br>Assessing lymph node metastasis:<br>51 [90]	Articles selected from 1995<br><br>Different MRI technologies (0,5 T; 1,5T; 3T; NA)<br><br>Mostly based on early-stages<br><br>Nodal regions subgroup analysis not performed
Tian et al., 2022 [85]	Yes	Yes	US	11	Assessing parametrial involvement:<br>62 [91]<br><br>Assessing stromal invasion:<br>84 [80]<br><br>Assessing lymph node metastasis:<br>52 [95]	Does not categorize early vs LACC<br><br>Large heterogeneity of studies<br><br>Nodal regions subgroup analysis not performed
He et al., 2022[98]	No	Yes	Conventional MRI, PET-CT	11	Assessing lymph node metastasis:<br>PET-CT 65 [93]<br>MRI 58 [91]	Does not categorize early vs LACC

		Majority of studies being retrospective<br><br>Nodal regions subgroup analysis not performed<br><br>Technological features insufficiently detailed

Supplementary Table S3: Overview of meta-analyses on radiological local and nodal staging in cervical cancer
